# Mental Health Survey of Social Entrepreneurs During COVID-19: A Study From Pakistan

**DOI:** 10.3389/fpsyt.2022.849085

**Published:** 2022-06-23

**Authors:** Nida Hussain, Baoming Li

**Affiliations:** ^1^Business School, Zhengzhou University, Zhengzhou, China; ^2^Yunus Social Business Center, Zhengzhou University, Zhengzhou, China

**Keywords:** social entrepreneurship, mental health, economic crisis, COVID-19, social change

## Abstract

**Background:**

COVID-19 is widely considered one of the worst pandemics in history, resulting in worldwide lockdowns, social isolation, unemployment, and economic recession. With the prolongation of COVID-19, numerous people experience stress, depression, and other mental health challenges. Recently, several studies have been documented in the literature on mental health issues among students (related to medical or other fields), teachers, medical personnel, and nurses in the wake of the COVID-19 pandemic. However, social entrepreneurs (SEs) have received insufficient attention. This study aims to conduct an online survey in Pakistan's five major cities to investigate more about the mental health status of social entrepreneurs.

**Materials and Methods:**

An online survey which included the Patient Health Questionnaire-9 (PHQ-9) and Generalized Anxiety Disorder-7 (GAD-7) was employed to collect data. The data analyses were carried out employing descriptive statistics, chi-square test, and multiple regression analyses.

**Results:**

A total of 840 social entrepreneurs from Pakistan participated in the survey. Among these, 366 (43.6%) were female and 474 (56.4%) were male. The findings revealed that 709 (84%) social entrepreneurs were suffering from depression symptoms, and 600 (80%) were suffering from anxiety symptoms. The majority of social entrepreneurs with depression (*N* = 546) and anxiety (*N* = 567) had mild and moderate stages. In addition, optional open-ended questions were asked from SE participants that help to understand their perception and response to the COVID-19 pandemic.

**Conclusion:**

The study concluded that several SEs in major cities of Pakistan were experiencing depression and anxiety symptoms. Based on data analysis, male SEs were highly suffering from depression and anxiety than female SEs. Limited resources and changes in customers' behavior were one of the major problems that lead SEs to depression and anxiety during the COVID-19 epidemic. In addition, SEs revealed that the lack of a social business execution policy is the most distressing factor for them. Therefore, a local government must take rigorous precautionary measures to prevent mental health issues among social entrepreneurs. Moreover, the Government of Pakistan needs to adopt supportive policies to assist social entrepreneurs in stressful circumstances.

## Introduction

In December 2019, the Chinese government first reported the “severe acute respiratory syndrome coronavirus 2” (SARS-CoV-2) also recognized as COVID-19 in Wuhan city, China. Thus, the government officially took initial steps for prevention and announced the lockdown (1). Furthermore, World Health Organization (WHO) took serious note of COVID-19 and announced it as a pandemic on March 11, 2020 (2). Initially, the rapid spread of COVID-19 shocked the entire ecosystem, and people around the globe were not prepared to deal with this kind of circumstances. Therefore, lack of awareness, no vaccination, and delay in social isolation caused massive losses almost everywhere. However, a total of 290, 959, 019 confirmed infectious cases and 5,446,753 deaths were reported to WHO as of January 04, 2022 (3).

Pakistan registered the first COVID-19 infected case on February 26, 2020 (4). The Government of Pakistan implemented strict actions for COVID-19 prevention; precautionary measures were defined to the general public about the predicted spread of COVID-19 under the guidelines of WHO. According to statistics, the first lockdown was implemented on 24 March2020, in all provinces of Pakistan (4). A total of 1,298,763 confirmed infected cases and 28,950 death cases were reported to the Ministry of National Health Services Regulation and Coordination of Pakistan as of January 04, 2022 (5). Due to the severity of COVID-19 cases, the Pakistani government extended smart lockdowns and the entire business community came to an end. Consequently, a surge in unemployment has been observed, leading the general public to various kinds of health issues, such as mental health problems (6, 7).

With the continuation of COVID-19, economies, societies, and individuals' lifestyles were influenced negatively (8). In addition, the exponential growth in physical and mental health challenges ruined the psychological wellbeing and damaged the quality of life of people around the globe (9, 10). One worst repercussion of COVID-19 is that it leads the general public toward depression, anxiety, and other mental health-related challenges (11, 12).

On the contrary, mental health is an intriguing area in the field of entrepreneurship. According to recent studies, few research scholars addressed the problems faced by entrepreneurs during COVID-19 (13, 14). For instance, Mustafa et al. (15) conducted a qualitative study to trace the social and mental obstacles faced by women entrepreneurs in Pakistan. Their study highlighted several reasons that women entrepreneurs suffer during COVID-19 lockdown which include social, cultural, economic, mental health, and lifestyle crises. These females also tried to divert their business model toward digitization. Moreover, he concluded that strong emotional support can help entrepreneurs during the COVID-19 crisis. This research suggested that entrepreneurs need to work on their social skill development to prevent depression and anxiety and they should interact with each other. Hence, practicing these skills will help them psychologically and they will feel comfortable with the surrounded community. In addition, Ruiz-Rosa et al. (16) investigate the social entrepreneurial intentions among students during the COVID-19 crisis. Their study disclosed that there was a decrease observed in the European University students toward the intention of social entrepreneurship in COVID-19.

A report, issued by the World Bank, delivered 5 weeks of training to test the hypothesis based on cognitive behavior therapy (CBT). The final result of the study shows a significant change in the behavior of small business owners, entrepreneurs, and other business persons in the conflict-affected areas in Pakistan (17). However, such training are highly needed during the COVID-19 pandemic, and problems related to entrepreneurial mental health need to be addressed appropriately (18). A report by Kings Business School of London discussed various aspects and challenges faced by entrepreneurs. This report mentioned that entrepreneurs are often known as lonely workers and secluded heroes. Therefore, they have to take care of their subjective and mental wellbeing (19).

Social entrepreneurs (SEs) often refer to individuals with problem-solving nature. SEs develop business models according to social needs that include solutions, assistance for society (20), and reinvesting revenue (21) in social causes. Autin et al. (6) formally define SE as an individual with an innovative idea, the ability to generate social value, and take risks in uncertain conditions can remain to transpire inside or beyond the commercial sector, non-government organization (NGO), and government sectors. Social entrepreneurs are widely recognized as social problem solvers. SEs are different from typical entrepreneurs, and they are more sensitive toward social challenges (22). They linked different opportunities with social issues that may be exploited through the development of products and services which result in the creation of social value. Furthermore, for SEs, success is not associated with profits, revenue, or finance but a positive social impact is considered the biggest success (23). Conversely, no guidelines for social business execution and no state-owned operating measures are clearly defined for SEs (24, 25). Nevertheless, SEs accept challenges for better social change. However, this process is associated with great mental and social pressure (26). The report, published by Weare3sixty (27), found that entrepreneurs in the United Kingdom (UK) were 70% to 77% affected by mental and physical health. Moreover, they were three times higher depressed than common UK citizens. Thus, we can say that SEs carried a strong influence of social pressure on their shoulders.

Interestingly, various social enterprises have been proposed to solve problems related to mental health. In September 2020, a UK-based social enterprise received a £75,000 grant to support their cause for “mental ill-health ‘amplified' by the fall-out of COVID-19.” According to CEO Martin Hogg, “We're anticipating a surge in referrals, but also more complex issues as mental health problems are amplified. If someone had anxiety before COVID-19, they may have managed with coping techniques. Those with anxiety see the world as a dangerous or difficult place, and the global pandemic has just confirmed what they always thought was true, so people with anxiety issues are particularly affected by this and may need more intense support.” Hence, they aimed to educate, be aware, and help UK citizens with several mental health problems that arise due to COVID-19 (28). Similarly, Yunus and Youth, a social enterprise center by Dr. Younus (Nobel Prize winner), has arranged an online webinar for social entrepreneurs. The discussion was to understand the difficulties faced by social entrepreneurs, including fighting against depression, anxiety, burn-out syndrome, and suicide, among others. Furthermore, 1/3 of the entrepreneurs mentioned that they had negative suicidal thoughts. During the webinar, they shared about their mental health challenges and debated how they turned those impediments into an achievement to build a better future (29). It is believed that during the COVID-19 pandemic, the isolation developed pressures and limitations as a triggering point among SEs (30).

A plethora of research articles is available on the impact of COVID-19 on country residents, students, and people associated with the medical profession. Moreover, the researcher also defines the grave influence of lockdowns, social isolations, and fear of viruses on their mental health (31–33). However, Ratten (18) has addressed the research gaps that problems faced by social entrepreneurs during COVID-19 have been overlooked. With this in mind, the present study aimed to evaluate the mental health challenges, such as depression and anxiety, faced by the social entrepreneurs of Pakistan. Consequently, this article presents pioneering research in the field of social entrepreneurial mental health in Pakistan during COVID-19.

## Materials and Methods

### Sample and Procedure

The nature of this study was cross-sectional, and data were collected through a convenience and snowball sampling technique. The target population was the social entrepreneurs (SE) from five major cities (Islamabad, Rawalpindi, Lahore, Peshawar, and Karachi) of Pakistan. Due to COVID-19, offline field surveys were challenging. Therefore, an online survey link was shared using various social media (Facebook, WhatsApp, and LinkedIn) platforms and some personal contacts. Hence, over 61.34 million (34.3%) Pakistani have access to the Internet, and 46 million of them use social media regularly (34). In addition, Napoleancat.Com (35) reported on their website that 37 million people use Facebook as a primary medium of communication and 6 million professionals are active LinkedIn users in Pakistan. Moreover, SE participants were requested to share questionnaire links with their fellows.

This cross-sectional study approached SEs from April to June 2021. Withal, during this phase, Pakistan was under the third wave (peak) of COVID-19 (36). In addition, mental health has been stigmatized by society in developing countries (37). Hence, approaching social entrepreneurs was difficult for collecting data because when they heard about the mental health survey, they resisted and hesitated for filling out such a questionnaire. In an attempt to collect data, we assured SEs that their response will be kept confidential. Thus, a survey was completed anonymously. SEs were informed about the research purpose that this research is developed to investigate the influence of COVID-19 on their mental health. For better understanding, we requested some open-ended questions about SEs perception of COVID-19 on their mental health and social ventures. The survey was developed using the free software Google Forms®.

A validity assessment of the questionnaire was conducted by two professors with relevant backgrounds. The bilingual expert utilized PHQ-9 (38) and GAD-7 (39). Urdu (the native language) translated and valid questionnaire and approved for the online survey. The accepted questionnaire (https://forms.gle/pLZG4iVnGR6ts3wj7) consisted of three sections. The first section was based on the demographic details of the participants. In demographic details, participants' gender, age, education, working experience, and association with operating industries were asked. In addition, they were asked whether they were affected or not by COVID-19 (tested positive or negative with coronavirus).

The second section was based on Depression (PHQ-9) and Anxiety (GAD-7) questions. Various scholars used PHQ-9 and GAD-7 to examine the psychological impact of the COVID-19 outbreak in Australia, China, Hong Kong, and Nepal (40–44). In addition, the PHQ-9 and GAD-7 were reported as an exception validity among Pakistani samples (45–47). Therefore, the following research used PHQ-9 and GAD-7 to identify the prevalence of depression and the prevalence of anxiety among social entrepreneurs in Pakistan.

Lastly, section three includes four open-ended questions (optional). (1) What affected you most when the lockdown was announced during the COVID-19 pandemic? (2) How did you manage mental and physical health challenges during the COVID-19 pandemic? (3) To survive, businesses have shifted their business strategies to a digital paradigm. How did you deal with this situation? (4) Do you believe your social business will be able to withstand this current pandemic?

This study follows WHO recommendations to decide the sample size (48). An online sample size calculator (49) identifies that approximately 567 or more surveys are required to have a 95% of confidence level within ±5% of marginal error. Consequently, we targeted to collect 567 responses from April to June 2021. However, due to electronic data collection, 840 samples were collected (95% of confidence level and ±3.24 marginal error) during this timeframe. The overall response rate was observed as 84% that include 366 (43.6%) females and 474 (56.4%) males. The description of the statistics of the sample is shown in Table 1. The final questionnaire has an overall internal consistency of optimal value of 0.81(Cronbach's alpha), hence, > 0.7 (50).

**Table 1:** Descriptives.

**Demographics category**	**Frequency**	**Percentage**
**Participant city**		
Islamabad	116	13.8%
Rawalpindi	140	16.7%
Lahore	195	23.2%
Peshawar	85	10.1%
Karachi	304	36.2%
**Participant gender**		
Female	366	43.6%
Male	474	56.4%
**Participant age profile (years)**		
18 to 20 years	208	24.8%
21 to 30 years	369	43.9%
31 to 40 years	111	13.2%
41 to 50 years	84	10.0%
Above 50 years	68	8.1%
		
**Participant education profile**		
School Level	138	16.0%
Collage Level	275	33.0%
University	215	26.0%
Other	212	25.0%
**Participant working experience**		
1 to 3 years	258	31.0%
4 to 6 years	200	24.0%
7 to 10 years	188	22.0%
Above 11 years	194	23.0%
**Participant operating industry**		
Health/Medicine Industry	260	31.0%
Food Industry	303	36.1%
Manufacturing Industry	73	8.7%
IT Industry	88	10.5%
Textile Industry	44	5.2%
Waste Industry	43	5.1%
Others	29	3.5%
**COVID-19 tested**		
Positive	638	76%
Negative	202	24%
**PHQ-9**		
None	131	16.0%
Mild	316	38.0%
Moderate	230	27.5%
Moderate- Severe	97	10.5%
Severe	66	8.0%
**GAD-7**		
None	240	27.5%
Mild	291	35.0%
Moderate	276	33.5%
Severe	33	4.0%

### Measures

#### PHQ-9

This study uses PHQ-9 known as Patient Health Questionnaire (51). This questionnaire is based on nine items that help to observe the total depression severity level (52). PHQ-9 is particularly used to monitor adolescent-sensitive behavior with assumed depression status (53). Thus, to measure the accurate frequency of SEs mental health status during the COVID-19, they were instructed about the appropriate way to answer the questions related to depression. PHQ-9 constitutes nine questions with responses ranging from “not at all,” “several days,” “more than half-day,” to “nearly every day.” The score mentioned in the answers is as follows: 0 points were assigned to not at all, 1 point was assigned to several days, 2 points were assigned to more than half-day, and 3 points were assigned to nearly every day. This point scale measured the total interpretation scores from 0 to 27, such as 0 to 4 as none with no depression (Level 0), 5 to 9 as mild level with a slight depression (Level 1), 10 to 14 as moderate depression (Level 2), 15 to19 as moderate-severe depression (Level 3), and 20 to 27 as severe depression (Level 4). Furthermore, the optimal point for sensitivity is 82% and specificity is 91% (54, 55). According to this study, the psychometric properties of the PHQ-9 in Pakistani populations have been confirmed (56). In the following study, Cronbach's alpha coefficient of the PHQ-9 was 0.82.

#### GAD-7

This study also used GAD-7 known as generalized anxiety disorder-7 to collect data (57). The following questionnaire consists of seven items that evaluate the total nervousness and anxiety issues. This tool enquires about the restless, worrying, easily annoyed, and other mental health problems (58). Therefore, the subjects were instructed to answer the questions related to the anxiety they experienced during COVID-19. GAD-7 consists of seven questions with responses ranging from “not at all,” “several days,” “more than half-day,” to “nearly every day.” The score mentioned in the answers is as follows: Not at all as 0 points, several days as 1 point, more than half-day as 2 points, and nearly every day as 3 points. This point scale measured the total interpretation scores from 0 to 21, such as 0 to 4 as none with no anxiety (Level 0), 5 to 9 as mild level with slight anxiety (Level 1), 10 to 14 as moderate anxiety (Level 2), and 15 to 21 as severe anxiety (Level 3), hence with the optimal point for sensitivity 90% and specificity 92% (59, 60). Hence, GAD-7 had 0.80 of Cronbach's alpha coefficient.

### Data Analysis

All statistical analyses were performed using R-studio (version 1.4). Sampling features (frequency and percentages) were assessed using descriptive frequency statistical analysis. Furthermore, the study opted Kolmogorov–Smirnov (K-S) test (61) to check the normality. K-S test is recommended for a larger sample size (N≥50) (62). Hence, null hypothesis states that *P* > 0.05 endorse continuous data are taken from normally distributed inhabitants. Thus, data in this study were normally distributed. Consequently, researchers (63, 64) implemented the chi-square testing method to better understand the significant difference between symptomatic and asymptomatic. In this study, SEs symptomatic and asymptomatic in terms of age, city location, gender, and operating industry have been identified. The chi-square test defines the relationship between perceived data and predictable data (65). Moreover, multiple linear regression was used to measure the scores on depression and anxiety and their odds ratios (OR) and 95% confidence intervals (95% Cl). Hence, the *P*-value of < 0.05 was considered statistically significant (66). All the reagent details are mentioned in Table 2.

**Table 2:** Association of demographic details with depression and anxiety.

	**Depression (PHQ-9)**					**Anxiety (GAD-7)**				
**Variable**	**Asymptomatic**	**Symptomatic**	***P-***	**OR**	***p- value***	**Asymptomatic**	**Symptomatic**	***P-***	**OR**	***p- value***
	**(≥0 and ≤ 4)**	**(≥5 and ≤27)**	***value***	**(95% CI)**		**(≥0 and ≤4)**	**(≥5 and ≤21)**	***value***	**(95% CI)**	
**Participant gender**										
Female	68 (18%)	298 (82%)	**0.118**	0.14 (-0.36 to 0.63)	0.68	100 (27%)	266 (73%)	**0.329**	0.11 (-0.48 to 0.47)	0.99
Male	63 (13%)	411 (87%)		*Ref*	*Ref*	140 (29%)	334 (71%)		*Ref*	*Ref*
**Age (years)**										
18 to 20 years	27 (13%)	181 (87%)		*Ref*	*Ref*	65 (31%)	143 (69%)		*Ref*	*Ref*
21 to 30 years	64 (17%)	305 (83%)		−0.17 (-0.55 to 0.36)	0.031	112 (30%)	257 (70%)		−2.25 (-3.88 to 6.05)	0.021
31 to 40 years	24 (22%)	87 (78%)	**0.046**	1.35 (-2.77 to 2.03)	0.045	26 (23%)	85 (77%)	**0.029**	3.71 (2.81-6.89)	0.035
41 to 50 years	9 (11%)	75 (89%)		0.53 (-1.43 to 3.38)	0.043	21 (25%)	63 (75%)		0.42 (-1.18 to 1.46)	0.46
50 years above	7 (7%)	61 (93%)		0.44 (-3.95 to 0.79)	0.033	16 (24%)	52 (76%)		1.48 (1.37-2.23)	0.027
**Participant city**										
Islamabad	40 (34%)	76 (66%)		*Ref*	*Ref*	37 (30%)	79 (70%)		*Ref*	*Ref*
Rawalpindi	32 (23%)	108 (77%)	**0.049**	0.05 (-4.17 to 0.34)	0.02	38 (27%)	102 (71%)		0.23 (-0.41 to 0.27)	0.04
Lahore	13 (9%)	182 (91%)		0.35 (-2.1 to 1.99)	0.011	55 (28%)	140 (72%)	**0.047**	1.89 (-1.91 to 3.56)	0.032
Peshawar	14 (17%)	71 (83%)		4.49 (-3.57 to 5.66)	0.037	26 (30%)	65 (70%)		2.92 (2.29-3.89)	0.081
Karachi	32 (11%)	272 (89%)		3.13 (1.22-4.97)	0.001	84 (28%)	220 (72%)		1.79 (1.68-6.75)	0.001
**Operating industry**										
Health/Medicine Industry	40 (15%)	220 (85%)		*Ref*	*Ref*	72 (28%)	188 (72%)		*Ref*	*Ref*
Food industry	43 (14%)	260 (86%)		2.23 (1.79−2.67)	0.001	86 (23%)	217 (77%)		2.17 (3.37−2.23)	0.011
Manufacturing Industry	21 (29%)	52 (71%)		0.80 (0.65−1.48)	0.028	19 (26%)	54 (74%)		2.22 (-1.62 to 3.11)	0.019
			**0.046**					**0.031**		
IT industry	12 (14%)	76 (86%)		3.35 (-4.73 to 4.02)	0.041	30 (34%)	58 (66%)		4.99 (-3.77 to 5.46)	0.022
Textile industry	4 (9%)	40 (91%)		1.53 (-1.63 to 2.48)	0.01	19 (43%)	25 (57%)		4.13 (-1.82 to 4.97)	0.039
Waste industry	6 (14%)	37 (86%)		0.44 (-4.95 to 0.89)	0.023	11 (26%)	32 (74%)		1.93 (-1.63 to 3.58)	0.034
Others	5 (10%)	24 (90%)		0.15 (-2.82 to 1.11)	0.052	3 (10%)	25 (90%)		0.44 (-5.95 to 1.89)	0.28

*N = 840*.

The last section of the questionnaire was kept optional. A total of 148 SEs responded to open-ended questions. Respondent replied in the English language. The thematic analysis carried out in this study elaborates the methodology of “reduction” (67). Reduction methodology sharpens, categories, eliminates, and assembles the qualitative data, to draw and verify “final” assumptions (68). The qualitative analysis was performed using QDA Miner (version 6.0). Hence, data were themed and coded according to the six-step method of thematic analysis (69). First, researchers familiarize themselves with collected data. Second, generated initial codes by reading transcripts. Third, developed themes from initial codes. Fourth, themes were reviewed again and developed new themes. Fifth, themes were defined clearly. Sixth, all the assumed codes and themes were write-up accordingly.

## Results

The following section is divided into two sections: the quantitative and qualitative parts.

### Quantitative Part

#### Descriptive Detail

A total of 840 responses were collected. The overall response rate was observed as 84% that includes (*n* = 366, 43.6%) females and (*n* = 474, 56.4%) males. For the respondents, 275 (33.0%) held a college-level education (12 years of education), 304 (36.2%) were from Karachi, 369 (43.9%) were 21–30 years old, and 303 (36.1%) were associated with the food industry. Further, 638 (76%) SEs were tested COVID-19 positive and 202 (24%) were negative. All the demographic details of participants are mentioned in Table 1.

#### Mental Health Status

The analysis of PHQ-9 and GAD-7 showed that the SEs of different genders were not significantly different in depression (*OR* = 1.4, 95% CI = −0.36 to 0.63; *p* > 0.05) and anxiety (*OR* = 0.11, 95% CI = −0.48 to 0.47; *p* > 0.05). Further, age between 21 and 30 years old highlighted a significantly associated with depression (*OR* = −0.17, 95% CI = −0.55 to 0.36; *p* < 0.05) and anxiety (*OR* = −2.25, 95% CI = −3.88 to 6.05; *p* < 0.05). For the different cities, results reveal that SE participants from Karachi scored higher in depression (*OR* = 3.13, 95% CI = 1.22–4.97; *p* < 0.05) and anxiety (*OR* = 1.79, 95% CI = 1.68–6.75; *p* < 0.05). The symptoms of depression (*OR* = 2.23, 95% CI = 1.79–2.67; *p* < 0.05) and anxiety (*OR* = 2.17, 95% CI = 3.37–2.23; *p* < 0.05) were noted to be significantly higher among SEs from food industry. However, the overall prevalence of depression was 42.98% and the prevalence of anxiety was 22.74%. All relevant details are mentioned in Table 2.

### Qualitative Part

The following section explored the open-ended questions. Nearly, all SEs reported numerous structural issues and challenges faced during the ongoing COVID-19 pandemic. They identify the uncertain situation they are still facing during this ongoing pandemic. During compiling transcripts, 86 were females and 62 were males. The majority of these were from Karachi city, and the ratio of females affected by depression and anxiety was higher than males in this qualitative part. The analysis of responses was further divided into five main themes: (1) Change in Customer Behavior, (2) Lack of Resources, (3) Mental Health, (4) Transformation in Business Model, and (5) Coping Mechanisms for Mental and Physical health. Lastly, throughout this section, RP refers to the response of participants with numbers (1, 2, 3…).

#### Change in Customer Behavior

The majority of SE respondents identify that they face financial crises during COVID-19. According to them, they were not able to sell products and services which damaged their revenue chart, such as respondent RP1 from the manufacturing industry mentioned, “*The phase in which we are currently living has been one of the most difficult phases in our lives. I don't know how to recover losses and bring my business on track. I lost plenty of customers.”*

Similarly, respondent RP13 from the food industry explains, “*I have seen a lot of tough situations in my life, but I have never felt as helpless as I did in this situation. At the beginning of the pandemic, people help and donate money to underprivileged people. Later, people start losing their jobs and they stop supporting our business. Now we don't have money to execute business like before.”*

Another respondent RP32 from the textile industry stated, “*Previously, customers used to place orders and they pay in credit. But now everything is in cash. Moreover, customers become more attentive to spending money. These rapid changes in business operations fear me.”*

The responses of above the respondents reveal that customers are more concerned about the value of money, commonly prefer to save money for their basic needs. Hence, one can say that variations in customer behavior were observed during this ongoing pandemic. Thus, this customer behavior leads SEs toward helplessness, hopelessness, and fearing feelings of discontinuing their business.

#### Reduction in Resources

As the financial chain was cut off due to lockdown, SEs were not able to pay bills, rents, and salaries to employees. It was also observed that numerous SEs expel their employee from work, such as a respondent RP28 from the textile industry mentioned, “*I have no option, but dismiss employees from business because I can't pay them salaries.”*

SE from the Waste industry respondent RP91 stated, “*Most of my workers collect waste products and brought it to the production house. However, due to lockdown and unavailable financial support now I can't pay them for collected waste.”*

Another SE respondent RP41 from the food industry described, “*Due to COVID-19 fear, most shops and markets remained closed. This creates a reduction in raw material supply. Even customers place an order but I don't have material to cook food according to their required demand.”*

However, the situation got more worsts when a few of SEs identified that they sold their office supplies to pay employee salaries. According to them, in a long-term scenario, they cannot keep these employees with them, such as respondent RP108 from the manufacturing industry alleged, “*I can't pay my employees because industries were closed. Consequently, I have to sell office furniture to pay their salaries.”*

Another response RP100 from the manufacturing industry stated, “*there is no clear policy from government and resource distribution supply chain is badly damaged due to lockdown. I don't know what should I do?”*

Following responses from participants expressed that the COVID-19 pandemic badly affected their social business. This pandemic created a market gap in form of not available resources mostly in form of financial resources. Thus, the COVID-19 pandemic made SEs compel to quit their employees. Moreover, no clear policy is also lacking to support SEs during the pandemic.

#### Mental Health

The prolongation of COVID-19 and its association with a lack of financial and human resources cause a huge mental challenge for SEs. Social entrepreneurs found them in a state of depression and anxiety to fulfill their requirements. According to SEs, recent survival in a market with an increase in prices (inflation) and an unstable economic situation is challenging. They feel depressed about the future which is based on thinking about “what will happen next.” Same as responded.

RP8 from the manufacturing industry mentioned, “*I feel like I am suffering from depression because I can't pay rent and I don't have any other option to close my unit.”*

Moreover, respondent RP15 from the waste industry revealed, “*I can't sleep at night. I kept on thinking about how to survive in the future with COVID-19.”*

SE from the IT industry, RP96 highlighted that mental health issues are not associated with the physically operating industry. Nevertheless, SEs from the IT industries are also facing numerous challenges, such as he mentioned, “*Before, COVID-19, I used to get the opportunity to go out and socialize with people which relaxed my mind. However, during the pandemic, I am only limited to my work and had no way out or no socializing. This situation made me depressed.”*

Hence, these responses from SE divulge mental health problems are usually associated with survival thinking. Plenty of SEs are considering the impact of COVID-19 might not allow them to survive in the long run. In addition, SE also identifies that continuous online operational activities with isolation lead them to depression and anxiety. However, few SE mentioned that they adopted technology to survive in the market. Contradicting to that few SEs were not ready to adopt the technology.

#### Transformation in Business Model

Due to lockdown and social isolation, plenty of businesses shifted their conservative business model to technology. Same as the number of SEs utilizing technology to survive in a pandemic. Many of SEs from the food industry stated that their way of work could not completely transfer to digitalization. They have to perform manually with available manpower. In this regard, RP140 from the food industry stated that “*food is cooked by humans not machines. We are not a technology-oriented nation. We have to perform most tasks by hand. I can't completely rely on technology.”*

The same goes for the waste industry, a responder used to make handbags from waste material. RP110 quoted that “*I work with the labor workforce in my production unit. We are known for handmade handbags. I can use technology to market my product but in production, but we have to work manually.”*

Contradicting, businesses associated with the IT and software-based were more relaxed during this phase. They were already using technology and providing their services. A respondent from the IT industry replied that “*I was familiar with the technology. Instead of operating from the office now, I work from home. COVID-19 only changed my working environment.”*

The responses from SEs show that COVID-19 brought opportunity and crisis at the same time. For a few SEs, this pandemic was not more than a disaster that lead them to close their business. On the contrary, it also provides opportunities for SEs to earn more. In addition, some SEs identify that their business nature does not support them to transfer their business model to technology. However, few SEs stated that they adopt technology as a coping mechanism.

#### Coping Mechanism for Mental and Physical Health

When the subjects were questioned on “how they cope with their mental and health issues?”, the majority commented that they mostly concentrate on religious and spiritual activities. A small minority focused on physical exercises and some stated that family support helps them in coping with mental and physical issues. Likewise, the response from SE of the health industry mentioned that “*I prefer to do exercise. To keep my body and mind active.”* Conversely, a respondent from another (education sector) talked about increasing religious and spiritual activities help to keep their mind calm. Responses are mentioned with codes and themes in Table 3.

**Table 3:** Coding and themes.

**Compiled responses**	**Codes**	**Themes**
•I was quite shocked due to the announcement of lockdown because we had to shut down the business. There was no way of income.	Less buying Saving money	Change in customer Behavior
•COVID-19 affected us financially. Everything shut down. Several businesses closed down in our industrial zone and customers are not buying products anymore.		
•I observed a drastic reduction in sales.		
•I feel customer behavior has changed and they have stopped buying our products.		
•COVID-19 pandemic makes people think before they buy any product.		
•I feel it's time to save money, this is apparent from customer behavior.		
•COVID-19 affected my business very badly as people are so scared about the financial situation, they are placing very limited orders.		
•COVID-19 has affected the entire world. In Pakistan, the business market is also influenced critically. Customers were so frightened initially that they were not ready to talk or place an order. Furthermore, the supply chain was affected due to social distancing.		
•People think they will be bankrupt. That's why they are buying only in cash. No advance payments are made these days.		
•I have reduced the number of employees working with me. I don't have money to pay salaries to employees, therefore, I have fired them.	Reduction in employees Reduction in finance	Reduction in resources
•I have sold some office equipment to pay the salaries to employees but now it's not possible anymore to keep an employee for the long run.		
•I am worried about how to pay bills, and how to pay salaries to employees. I think I need to start downsizing my company.		
•Government allow us to reopen business but follow SOPs. The nature of my business is to directly communicate with clients. But now, clients don't avail of any of my services due to fear of COVID-19. That's why I ask my employees to be quit.		
•I think the main issue is the financial crisis because we have no clients and there is no way of income. Day by day, everything is getting too expensive which is hard for us to afford.		
•During COVID we experienced a severe downfall in business. We were not in a position to pay our staff members. Eventually, we had to fire them. To sort out the crisis, we had to sell the furniture in our offices.		
•I feel if the situation remains the same, I will not be able to survive, it depresses me. The economy is getting worse and the rising inflation has caused huge damage to my business. It seems impossible to survive. I am in depression.	Hopeless Depression Anxiety	Mental health
•1st wave of COVID-19 affected us so badly that we have lost customers and money. It has affected me mentally and physically.		
•No, I don't think, that I will survive because of a lack of resources now. I am trying my best to run my business. However, I feel that I will shut off all my hard work one day.		
•I got sacred when the government declared the lockdown due to COVID-19. But, we had not thought that the lockdown would last for so long. It engulfed the whole year. Isolation and restriction disturb me mentally.		
•Every night I think about what will happen tomorrow. Which stresses me so much.		
hline •The sudden announcement of a lockdown does not give us a chance to shift our business to digitalized platforms.	Technology acceptance Technology implementation	Transformation in business model
•I did not work online during the lockdown and ongoing COVID-19 pandemic, as my business is concerned with physical appearance. I cannot perform online tasks.		
•Doing business on digital platforms was quite difficult because I don't possess the knowledge of social media and other digital platforms to do business. But now, I have learned the business techniques and related social media apps usage. It becomes easy.		
•COVID-19 affected businesses on a large scale. We were unable to pay wages to the workers. The material was not available for production. Prices soared high in a lump sum and it was a great loss for us.		
•I was familiarized with the technology. With time, I tried to improve my business and updated it. I developed my website and a Facebook page. I was highly active on Instagram, used WhatsApp and also digital marketing to upscale my business and track customers.		
•I recently started a business and made a plan to shift business to a digital platform within 3 years. However, COVID-19 allowed me to shift business before my forecasted time.		
•Most businesses joined online platforms and became digitalized because people were restricted to staying at home. For the last 2 months, I am working with my team to digitalize my business completely.		
•I practice religious activities regularly to relax my mind.	Follow religion Exercise	Coping mechanism for mental and physical health.
•Nothing is better than following the preaching of Islam. It changes my mind.		
•I think five times prayers in a day are enough for me to cope with a difficult situation.		
•I do mediation and exercises to calm my nerves.		
•I think about the positive aspects of life.		
•I do yoga on regular basis to cope with mental and physical health.		
•Family is my stress relief. I spend time with them.		
•I have supportive family and friends, they help me in a difficult times.		

#### Visual Text Interpretation

**Figure 1:**
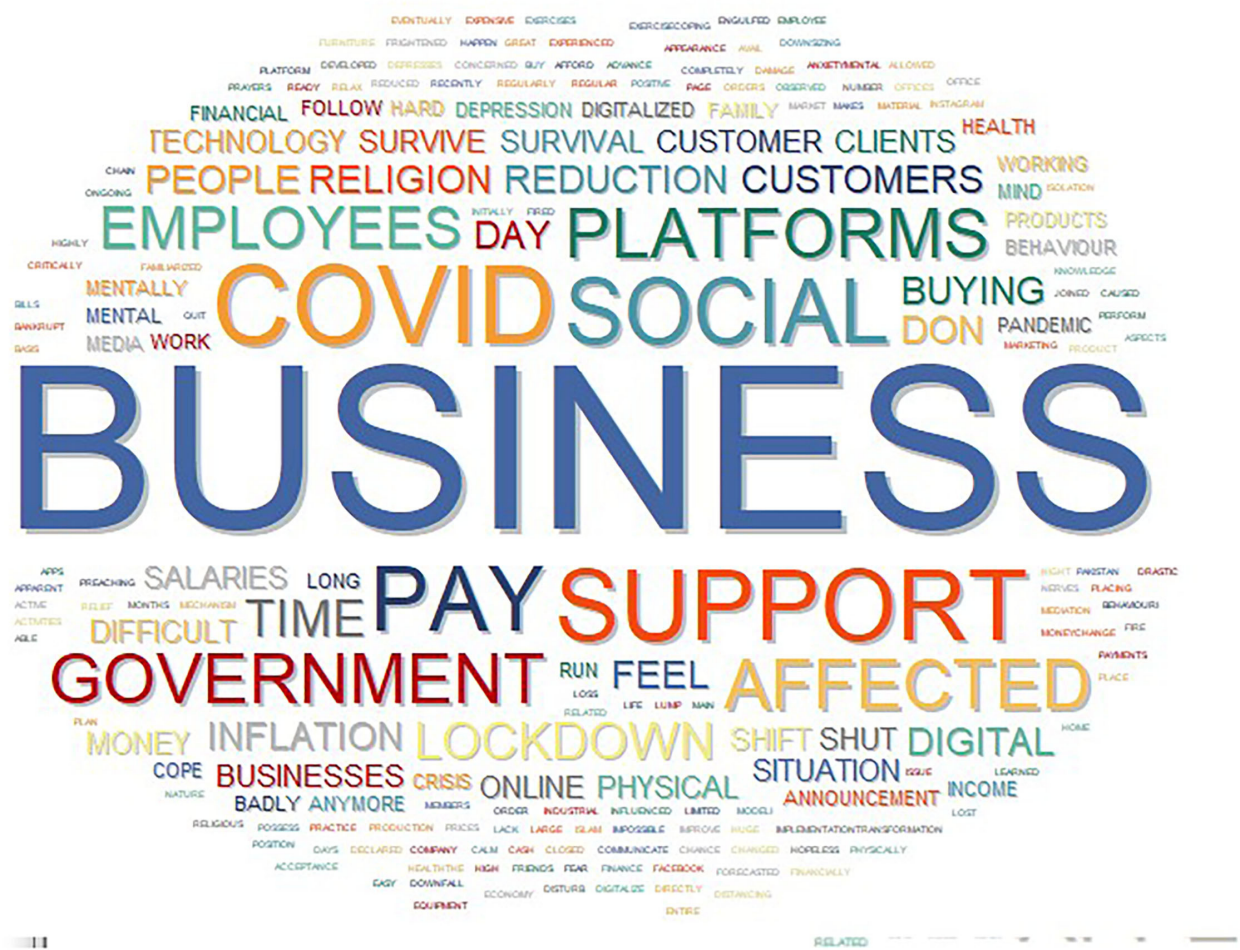
Visual words cloud.

This study creates a word cloud to interpret the text visually. In Figure 1, the most prominent words in the study are illustrated. These words include Social Business, COVID, Pay, Support, Government, customer behavior, mental health, reduction in resources, employees, and entrepreneurship. Compared to other factors, these words' prominence in the study suggests these factors played a critical role. In addition, it interlinks all the factors, challenges, and objectives of the study, while the words, inflation, survival, pay, and support appear in less noticeable colors as a lower order terms. Supplementary, the words, religion, business, platforms, social support, and other problem seemed as minor footings illustrating the influence of these independent features on the relationship between the COVID-19 pandemic, mental health, and social entrepreneurship.

## Discussion

Previous studies have concentrated on the mental health status of students, frontline medical staff, and other individuals (70, 71). However, to the best of our knowledge, limited research evidence has been found on the mental health status of social entrepreneurs in Pakistan. The purpose of this study was to evaluate the mental health status of SEs in Pakistan during COVID-19. Our study provides additional support to social entrepreneurial literature in terms of their mental health conditions during COVID-19. Patient Health Questionnaire-9 (PHQ-9) and Generalized Anxiety Disorder-7 (GAD-7) questionnaires were used to identify the SEs mental health. Hence, our results revealed mild-to-moderate levels of depression (*n* = 546, 64.5%) and anxiety (*n* = 567, 67.7%) among the survey participants. Moreover, COVID-19 tested positive individuals were highly affected by depression and anxiety. Hence, we can relate this result to Hayat et al. (56) study, which concluded that psychological disorders are more common in the general population of Pakistan during the COVID-19 pandemic.

Data demonstrate that 298 females and 411 males were identified as being depressed, while 266 females and 334 males were reported as being anxious. Hence, there were no significant differences in the prevalence of depression and anxiety in terms of gender which verify that psychologically both genders' SEs were affected by the COVID-19 pandemic. As stated earlier, previous work has only focused on the medical staff, students, and teachers for identifying depression and anxiety. Using previous work as a reference, a plethora of researchers has been documented in the literature (15, 47, 70, 72, 73) identifying a significant difference among gender. Their findings concluded that females are most likely to suffer from depression and anxiety during the COVID-19 pandemic in Pakistan. In contrast to previous research, our findings revealed no significant difference in the prevalence of depression and anxiety among SEs gender.

This study also reveals that diverse ages of SEs had a significant difference in the prevalence of depression and anxiety. A large portion of SEs between the age of 21 to 30 years old has participated in the survey. Age has been observed as a significant factor in our research. Likewise, research by Qiu et al. (74) and Tull et al. (75) indicated a strong age connection with depression in China and United States. However, variation in results could be due to differences in the perspective and sample population.

An unexpected finding in our study shows significant differences in the prevalence of depression and anxiety in terms of five major cities in Pakistan. SE participants from Karachi were observed as a highly psychologically affected city. Karachi is a metropolitan city in Pakistan. Mostly, social entrepreneurs initiate their social businesses in this port city (76). Consequently, the ratio of participants and severity of depression and anxiety is higher as compared to other cities (73). Our result is consistent with previous results (77), which identify that there is a significance in the prevalence of depression and anxiety associated with different cities.

Likewise, the result also discloses that operating industries had significant differences in the prevalence of depression and anxiety. Here, it is noteworthy that SEs associated with the food industry were highly affected among other different operating industries. However, research by Khan et al. (78) identifies that the hospitality industry in Pakistan has been badly affected by COVID-19. Therefore, all the related businesses, like dining, restaurants, and picnic spots remained closed. Thus, it was discovered that the severity difference among age, city, and operation was high at a mild and moderate level of depression and anxiety. Few of SEs had moderate-severe and severe levels of depression and anxiety.

In the comment section of the survey, we enquired open-ended questions. Some SEs mentioned their perception and impact of COVID-19 on their mental health and social venture. Surprisingly, SE stated that they regularly feel depression, stress, worry, and anxiety. The majority of SE were depressive about the future. They used to think that what will happen if their social venture will no longer survive in such a turbulent environment of COVID-19. Several SEs feel motivated during the initial days of COVID-19, they specified that people tried to support their social venture by giving donations. It was also observed that in a pandemic crisis, the behavior of investors to invest in social enterprises was highly volatile (79). However, extended lockdown, economic crisis, and other related obstacles reduce the financial support day by day. Several SEs reasoned that they reduced the number of employees to support their business. The furloughing scheme was introduced for employees, unfortunately, due to the large informal economy of Pakistan; it was not manageable for entrepreneurs in Pakistan (19). Prior studies have shown that those business owners who went bankrupt and are unemployed during COVID-19 face more severity in mental problems (12).

Apart from these aforementioned reasons, technology adoption is also a cause of their worry. Old-aged SEs feel tranquil in the traditional way of operating ventures. It seems likely that old-aged SEs are not ready to adopt the technology. In addition, few SEs stated that the nature of their business is not suitable for shifting business to digitalization. Besides, few SEs stated that their businesses are associated with the social wellbeing of society but the government is not supporting them. Hence, a total of 19% of entrepreneurs located in Pakistan applied for government support during the COVID-19 pandemic (19). Lastly, SEs believed that spiritual and religious influence helps them to remain calm in such a pandemic situation. They used religion and spirituality to cope with their mental and physical health. It is assumed that religion plays a pivotal role in the life of entrepreneurs in times of the COVID-19 crisis (80).

### Limitation and Future Direction

In general, entrepreneurs, especially social entrepreneurs, are a special group that should not be ignored during the COVID-19 crisis. Not only social entrepreneurs in Pakistan but also worldwide social entrepreneurs should be given specific attention. This study infers that local governing bodies need to adopt various methods to avoid, diagnose, and deal with the challenges related to mental and physical problems of social entrepreneurs during enormous scale stressors (e.g., the epidemic of infectious disease, economic crisis, and natural disasters). Prevention of serious consequences needs proper effective screening. Professional and solution providers for mental health issues should assist social entrepreneurs to prevent themself from high risk of emerging mental health complications.

Finally, some potential weaknesses need to be considered. First, this study was a cross-sectional study, data were collected from April to June 2021 (third wave), and the investigation of the psychological alterations of SEs across different phases of COVID-19 would provide a better understanding of the impact of the illness. Second, semi-structured open-ended investigative interviews utilizing PAQ-9 and GAD-7 were self-assessed, which might introduce a biased effect. Further, professional methods are required to understand the in-depth study. Third, SEs from big urban cities of Pakistan participated in the survey, and the possible reason is the availability of the Internet. Further studies, which take rural SEs into account, will need to be undertaken. Fourth, study subjects in the Pakistani context. We propose that further research should be performed in different countries (including China, Russia, and India). Fifth, past and post prevalence of depression and anxiety among SEs were not discussed. Finally, the present survey did not discuss the severe level of SEs who suffered from mental illness before the COVID-19 pandemic.

## Conclusion

This study aims to identify how different levels of depression and anxiety affected Pakistani social entrepreneurs during the pandemic. For this purpose, a mixed research method was adopted. The results of the data analysis revealed that a significant number of SE participants in the study experienced depression and anxiety. The study's findings corroborate the helplessness and hopelessness of SEs in executing their businesses in the market. The primary impediments were changes observed in customer behavior, lockdowns, social isolation, and limited resources that lead SEs toward depression and anxiety during the current COVID-19 pandemic. It is worth noting that the majority of SEs mentioned that exercise, family support, spiritual practices, and yoga were effective techniques they adopted to fight against depression and anxiety. Furthermore, technology adoption was a business survival strategy for some SEs.

Depression and anxiety are significant mental health symptoms among social entrepreneurs and may become more prevalent as we attempt to reintegrate socially. Thus, it is suggested that the Pakistani Government should support social enterprises that deal with mental health issues to assist the social entrepreneurs in such challenging circumstances. Unlike the Pakistani Government, the United Kingdom and other countries have launched a variety of educational training and webinar online for social entrepreneurs to educate them on mental health issues (28, 29). Similarly, the government and policymakers of Pakistan should take precautionary measures to address the challenges faced by social entrepreneurs.

## Data Availability Statement

The raw data supporting the conclusions of this article will be made available by the authors, without undue reservation.

## Ethics Statement

The research is approved by Professors Committee from Business School, Zhengzhou University, China. This study is not used for any medical and commercial purpose. It is mainly conducted to identify the mental health issues faced by Social Entrepreneur of Pakistan by survey method. We reached Social Entrepreneur using social media and personal contact. With their agreement anonymous data was collected. Therefore, written informed consent for participation was not required for this study in accordance with the national legislation and the institutional requirements.

## Author Contributions

NH and BL contributed to the design and conduct of the study. NH conducted the statistical analysis, interpretation of the data, and critically reviewed the important intellectual content. BL supervised the whole process and reviewed manuscript writing. Both authors contributed to the article and approved the submitted version.

## Conflict of Interest

The authors declare that the research was conducted in the absence of any commercial or financial relationships that could be construed as a potential conflict of interest.

## Publisher's Note

All claims expressed in this article are solely those of the authors and do not necessarily represent those of their affiliated organizations, or those of the publisher, the editors and the reviewers. Any product that may be evaluated in this article, or claim that may be made by its manufacturer, is not guaranteed or endorsed by the publisher.
